# Investigating the different domains of environmental knowledge acquired from virtual navigation and their relationship to cognitive factors and wayfinding inclinations

**DOI:** 10.1186/s41235-023-00506-w

**Published:** 2023-08-02

**Authors:** Veronica Muffato, Laura Miola, Marilina Pellegrini, Francesca Pazzaglia, Chiara Meneghetti

**Affiliations:** 1 Department of General Psychology, University of Padova, Via Venezia 8, 35131 Padua, Italy; 2 Interuniversity Research Center in Environmental Psychology (CIRPA), Rome, Italy

**Keywords:** Cognitive abilities, Navigation, Landmark, Location knowledge, Path knowledge

## Abstract

When learning an environment from virtual navigation people gain knowledge about landmarks, their locations, and the paths that connect them. The present study newly aimed to investigate all these domains of knowledge and how cognitive factors such as visuospatial abilities and wayfinding inclinations might support virtual passive navigation. A total of 270 participants (145 women) were tested online. They: (i) completed visuospatial tasks and answered questionnaires on their wayfinding inclinations; and (ii) learnt a virtual path. The environmental knowledge they gained was assessed on their free recall of landmarks, their egocentric and allocentric pointing accuracy (location knowledge), and their performance in route direction and landmark location tasks (path knowledge). Visuospatial abilities and wayfinding inclinations emerged as two separate factors, and environmental knowledge as a single factor. The SEM model showed that both visuospatial abilities and wayfinding inclinations support the environmental knowledge factor, with similar pattern of relationships in men and women. Overall, factors related to the individual are relevant to the environmental knowledge gained from an online virtual passive navigation.

**Supplementary Information:**

The online version contains supplementary material available at 10.1186/s41235-023-00506-w.

## Significant statement

Today, learning environmental information through devices showing paths and landmarks is a common and essential daily experience. This study showed that when individuals learn about a path passively through video of environments, they form an integrated and interconnected understanding of landmarks, locations, and the path itself. This ability is supported not only by the individual's cognitive abilities, but also by their beliefs about wayfinding. This notion is significant for everyday life as it highlights that improving visuospatial abilities and increasing positive wayfinding inclinations can help individuals better understand and remember their surroundings, even when they are not actively navigating the environment.

## Introduction

### Spatial navigation

Navigation is an essential, but complex everyday activity during which we gain environmental knowledge from a first-person viewpoint. When navigating an environment, we produce a spatial representation (Wolbers & Hegarty, 2010, or cognitive map; Tolman, 1948) with features that can be assessed using various tasks. Nowadays, the learning of a path by navigation and its recall are largely examined using virtual environments (VE) that can serve as a good approximation of real environments to explore (Richardson et al., 1999). Individuals can acquire spatial information from both active and passive navigation. Passive navigation is less effective in providing environmental knowledge than active navigation is (Chrastil & Warren, 2012, 2013, 2015; Do et al., 2021; Meade et al., 2019). Despite this, passive navigation is still important in everyday life, where it is common to learn about environments passively, such as when riding public transportation or as a passenger in a car, and then having to navigate back alone using a different means of transportation or way to orient oneself. The present study investigates people’s ability to learn a path passively in a VE presented on a desktop computer screen. The accuracy of path learning was tested using various types of environmental knowledge while also investigating their relationship with human factors such as visuospatial abilities and inclinations.

When learning an environment through navigation, people acquire spatial information from a first-person point of view that emphasizes an egocentric frame of reference, where the body serves as the primary reference point. However, when encoding and storing this information in memory, a combination of egocentric and allocentric representations can be done (Burgess et al., 2006; Ladyka-Wojcik & Barense, 2021). Therefore, various types of knowledge can be obtained after learning through navigation. According to well-established models of navigation (Golledge, 1999; Siegel & White, 1975; Wiener et al., 2009), this knowledge includes: (a) knowledge about points in space (also called landmark knowledge), (b) knowledge about sequences of points or paths (also called route knowledge), and (c) knowledge about areas or the spatial relationships between at least two points (also called survey knowledge).

Claessen and van der Ham (2017) recently went beyond this three-factor model (of landmark, route and survey knowledge) to propose a new classification of the spatial domains of knowledge starting from an assessment of individuals with neuropsychological navigation impairments. They found a functional dissociation between knowledge of landmarks, locations and paths, inasmuch as these individuals’ different neuropsychological impairments could affect one or more of these types of knowledge. Specifically, their classification suggests that navigation ability demands: (a) knowledge about landmarks, involving the ability to recall the elements present in an environment, which can be assessed with free landmark recall or landmark recognition tasks; and (b) knowledge about their locations, as seen in observer-based mode (location-egocentric knowledge; assessed, for instance, with egocentric pointing tasks) and in environment-based mode (location-allocentric knowledge; assessed, for example, with tasks that involve allocentric pointing or positioning single landmarks on a map). The classification thus distinguishes between two frames of reference, one egocentric (landmark-to-subject relations; assessed with route direction tasks or tasks that involve arranging landmarks in order, for instance), the other allocentric (landmark-to-landmark relations; assessed with sketch map drawings or shortest path finding tasks, for example) as a large body of research on spatial memory has previously suggested (Burgess, 2006; Iachini et al., 2023; Mou et al., 2006; Starrett et al., 2019, 2022; Starrett & Ekstrom, 2018; Zhang et al., 2014; Zhong & Kozhevnikov, 2016) and its development with age (Colombo et al., 2017; Ladyka-Wojcik & Barense, 2021; Ruggiero et al., 2016). The classification of Claessen and van der Ham (2017) also envisages the demand for: (c) knowledge about paths linking landmarks, considered both as a succession of elements encountered (path-route knowledge), and as an array of elements as seen on a map (path-survey knowledge); the classification thus also distinguishes between the survey (bird’s eye view) and route (observer’s point of view) perspectives (Taylor & Tversky, 1992). This model is partially in line with the one proposed by Wiener et al. (2009), that postulates a distinction between landmark or route/egocentric knowledge (based on remembering information from a person’s point of view) and survey/allocentric knowledge (based on remembering landmark-landmark information). It should be specified that knowledge in spatial mental representation is not necessarily structures with accurate metrical and coordinate metrical system, as postulated in the traditional concept of cognitive map (as proposed by Tolman, 1948), but instead, there is the notion of cognitive graph knowledge (Chrastil & Warren, 2014, 2015; Peer et al., 2020, 2023; Warren, 2019; Warren et al., 2017) that refers to the representation of the environment as a network of paths connecting nodes, that is, the places, without Euclidean information. In sum, navigation models propose various forms of environmental knowledge.

Given the multiple types of environmental knowledge gained from navigation, multiple tasks should be used in research investigating the functionally dissociable components of such knowledge (van der Ham et al., 2020). Very few studies examined all the different types of knowledge and how they are related, however (Muffato et al., 2022; van der Ham et al., 2020); and when they did, the focus was on age-related decline. Findings have shown that all types of environmental knowledge decline with age after passively learn from navigation (van der Ham et al., 2020), and particularly when tested in location-allocentric and path-survey modes in real environment learning (i.e. active learning, Muffato et al., 2022). Apart from age, however, there are other human factors relevant to how we learn environmental information from navigation. Previous research found that variability in the performance of tasks assessing different types of knowledge can relate to visuospatial factors (Allen et al., 1996; Ishikawa & Montello, 2006; Meneghetti et al., 2021; Weisberg et al., 2014). It therefore seems worth examining whether and to what degree visuospatial factors affect how different types of knowledge are acquired from navigation, especially passive navigation, which has received less attention.

### Spatial navigation and visuospatial factors

Visuospatial factors play a part in spatial learning (He et al., 2021; Hegarty et al., 2006; Ishikawa, 2022; Weisberg, et al., 2014). The term visuospatial factor covers a broad and heterogeneous set of aspects. One concerns visuospatial abilities, i.e. the cognitive skills used to generate, retain and manage abstract visual images (Lohman, 1988). These in turn include a subset of skills (as reviewed in Hegarty & Waller, 2005; Linn & Petersen, 1985), such as mental rotation (Linn & Petersen, 1985)—which can be separated (Hegarty & Waller, 2004) into: (i) object rotation, consisting in rotating an object in the mind’s eye, as measured with the Mental Rotations Test (MRT; Vandenberg & Kuse, 1978); (ii) and subject rotation, which involves adopting different views based on a subjective mental rotation, as measured with the Object Perspective-Taking test (OPT; Kozhevnikov & Hegarty, 2001). The two types of rotation ability are related (Hegarty & Waller, 2004), and both are involved in recall accuracy after learning from navigation; this involvement has been detected for location and path knowledge (e.g., in route repetition vs. shortcut tasks [Pazzaglia et al., 2018]; in map drawing, distance and direction estimations [Hegarty et al., 2006]; and in pointing tasks [Kozhevnikov et al., 2006]). Some studies consider visuospatial factors in terms of processing abilities, such as visuospatial working memory (VSWM), which enables us to retain and process visuospatial information (Logie, 1995). VSWM has been found related to learning from navigation too, using various tasks assessing location and path knowledge (route direction task [Garden et al., 2002]; direction estimations and shortcut tasks [Labate et al., 2014]; route repetition, map drawing, pointing tasks [Muffato et al., 2020]). When VSWM and mental rotation are both examined at the same time—considering the single contribution of each one (Allen et al., 1996; Meneghetti et al., 2016) or grouping them into a single factor (Hegarty et al., 2006; Pazzaglia et al., 2018) – they are found related to recall accuracy after learning from navigation.

Visuospatial factors also involve personal inclinations – which can be assessed with questionnaires—in terms of wayfinding attitudes and preferences (Meneghetti et al., 2021). Such questionnaires include various self-assessments, and mostly concern perceived sense of direction (De Beni et al., 2014; Hegarty et al., 2002; Pazzaglia & Meneghetti, 2017), or perceived navigation ability (He & Hegarty, 2020), preferred environment representation mode (as survey-like or route-like; Lawton, 1994; Pazzaglia & Meneghetti, 2017), positive attitudes to exploring places (pleasure in exploring, Meneghetti et al., 2021), spatial anxiety (Lawton, 1994), and spatial self-efficacy (our faith in our ability to cope with a wayfinding task; Pazzaglia & Meneghetti, 2017). Some studies focusing only on the effects of wayfinding inclinations found that a good navigation performance was related to low levels of spatial anxiety (assessed with a pointing task; Lawton, 1994), a strong sense of direction (path finding task [Hund & Nazarczuk, 2009]; pointing task [Labate et al., 2014]), more attitudes to exploring (shortcut finding task [Pazzaglia et al., 2017]; route repetition task [Muffato et al., 2019]), and greater self-efficacy (shortcut finding task [Pazzaglia et al., 2017]; pointing and map task (Miola et al., 2021). In short, a contribution of wayfinding inclinations seems to be detectable in recall accuracy after learning from navigation in terms of both location and path knowledge. The various wayfinding inclinations are related to one another (De Beni et al., 2014), and can be grouped into a single factor that has a role in predicting environmental knowledge (Meneghetti et al., 2021).

When wayfinding inclinations and visuospatial abilities were considered at the same time, they both supported spatial knowledge after learning from navigation, for both location, i.e. location-egocentric and allocentric knowledge (path-integration task [Muehl & Sholl, 2004]; pointing task [Muffato et al., 2022]; pointing task [Weisberg et al., 2014]), and path, i.e. path-route and path-survey knowledge (route repetition and sketch map drawing [Pazzaglia et al., 2018]; pointing, distance estimation, and map drawing [Hegarty et al., 2006]; route repetition [Muffato et al., 2019]; wayfinding task [Münzer & Stahl, 2011]; route repetition, shortcut finding, map drawing [Meneghetti et al., 2021]). Landmark knowledge has been less investigated than location or path knowledge, but there is some evidence of it correlating with visuospatial abilities too (Muffato et al., 2022). Muffato et al. (2022) were the first to investigate all the types of knowledge (landmark, location-egocentric, location-allocentric, path-route, path-survey) in relation to visuospatial factors in young, middle-aged and older adults. They found that, after learning environmental knowledge from navigation, VSWM was related to landmark and location-allocentric knowledge, and both VSWM and wayfinding inclinations were related to path-route and path-survey knowledge (after accounting for the role of age).

Taken together, the above findings offer fresh and encouraging evidence of various associations between multiple visuospatial measures and environment learning from navigation. That said, it is still impossible to draw any clear conclusions regarding the influence of visuospatial factors—simultaneously considering several visuospatial abilities and wayfinding inclinations – on how different types of spatial knowledge (Muffato et al., 2022; van der Ham et al., 2020) are acquired by navigating a VE. This issue deserves to be further investigated.

### Aim of the study

The aim of the present study was to examine the role of human factors, both visuospatial abilities and wayfinding inclinations, on the various types of spatial knowledge gained from exposure to a path passively presented in a VE, considering all the various types of environmental knowledge – landmark, egocentric and allocentric location, path route and survey knowledge – and their factorial structure.

A sample of young people was assessed on their visuospatial abilities (with a visuospatial working memory test, a mental rotation test and a perspective-taking test) and wayfinding inclinations (recording their self-reported sense of direction, attitude to orientation tasks and spatial self-efficacy). Then they learned a virtually-navigated path and were tested on their landmark knowledge (with a free recall landmark task), and their location-egocentric and location-allocentric (with egocentric and allocentric pointing tasks), path-route (with a route direction task) and path-survey knowledge (with a sketch map drawing task (presented in random order). The tasks were chosen to be similar to those used in previous studies considering all types of spatial knowledge gained from navigation (Muffato et al., 2022; van der Ham et al., 2020).

First, we ascertained the factor composition of visuospatial factors and the various types of knowledge. We expected visuospatial abilities and wayfinding inclinations to constitute two distinct factors (Meneghetti et al., 2021; see also Hegarty et al., 2006). We also investigated: whether each domain of spatial knowledge gained from navigation can be considered separately, given that these different outcome measures have been found dissociated (van der Ham et al., 2020); whether they can be grouped into landmark, location, and path knowledge (Claessen & van der Ham, 2017); whether they represent the three main types of knowledge, landmark, egocentric/route and allocentric/survey knowledge (Wiener et al., 2009); or whether the spatial navigation domains actually constitute a single factor (Hegarty et al., 2006; Weisberg et al., 2014), although previous research did not consider all the domains of spatial knowledge (van der Ham et al., 2020).

After testing the factor composition of the visuospatial factor/s and spatial knowledge factor/s, we examined how visuospatial abilities and wayfinding inclinations relate to environmental knowledge gained after passively navigating a VE. We expected to find both visuospatial abilities (mental rotation) and wayfinding inclinations (as previously found with sense of direction; Hegarty et al., 2006) related to performance in an environment recall task after learning about a VE from a video (as in Hegarty et al., 2006; Meneghetti et al., 2021). This would broaden our understanding of all the different types of spatial knowledge gained from navigation (as recently suggested by van der Ham et al., 2020) and how they relate to each other (factor composition). We explored whether there might be a noticeably different degree of involvement of visuospatial abilities and/or wayfinding inclinations as a function of the type of spatial knowledge task (testing each type of knowledge separately, and using a single- or three-factor composition). It may be that tasks assessing the recall of knowledge in a format similar to the learning condition (location-egocentric knowledge in the pointing task; path-route knowledge in the route direction task) might place a lesser burden on visuospatial abilities and/or wayfinding abilities than tasks that involve switching from an egocentric (in learning) to an allocentric approach (testing location-allocentric knowledge), and from a route (in learning) to a survey mode (testing survey knowledge), which could be more resource-consuming (as suggested by Meneghetti et al., 2021; Muffato et al., 2020).

## Method

### Participants

The study involved 270 young adults (145 females) from 20 to 40 years old (*Mean* age = 25.48, *SD* = 6.06). The sample size was calculated assuming at least 5 observations for each parameter of the structural equation model (Bollen, 1989). Participants were recruited by word of mouth or were students recruited in exchange for course credits. The Ethical Committee for Psychological Research at the University of Padova approved the study (univocal number: 0AE7DEE5519A7DFB70058638C8D23227). All participants were informed about the purposes of the study and gave their informed consent in accordance with the Declaration of Helsinki (World Medical Association, 2013). No participants were excluded.

### Materials

#### Session 1: visuospatial abilities and questionnaires

#### Visuospatial tasks

*Jigsaw Puzzle Test* (JPT, De Beni et al., 2008; see also Richardson & Vecchi, 2002; original version reliability *r* = 0.83). This VSWM task comprises up to 18 puzzles (two for each level of difficulty, ranging from 2 to 10 pieces) that must be completed mentally, without moving the pieces. Participants must solve both the puzzles on a given level of difficulty to proceed to the next level. The score corresponds to the sum of the correctly-solved puzzles (score 0–18).

*Short Mental Rotations Test* (sMRT, De Beni et al., 2014; adapted from Vandenberg & Kuse, 1978)*.* This consists in finding two of four objects (3D assembled cubes) that match a target object in a rotated position (10 items; time limit 5 min). The score corresponds to the number of correct answers (score 0–10; Cronbach's *α* = 0.72, current sample).

*Short Object Perspective-taking Test* (sOPT, De Beni et al., 2014; adapted from Kozhevnikov & Hegarty, 2001). In this test respondents have to imagine standing at one object in a layout comprising 7 objects, facing another, and pointing towards a third. Directions are indicated by drawing an arrow from the center of a circle to its perimeter (6 items; time limit 5 min). The score corresponds to the mean angular difference between the correct answers and the answers given (score 0–180°; Cronbach's *α* = 0.66, current sample).

#### Wayfinding inclinations questionnaires

*Spatial self-efficacy scale* (SSE; Pazzaglia et al., 2017)*.* This assesses how capable people feel when performing environmental spatial tasks, such as finding the right path in an unfamiliar environment (8 items). Each item is rated on a 6-point Likert scale (1 = not at all to 6 = very much) and the sum is calculated (score 8–48; Cronbach's *α* = 0.85, current sample).

*Attitude toward orientation tasks scale* (De Beni et al., 2014). This scale assesses an individual’s pleasure in exploring (e.g., “I like to find new ways to reach familiar places”) (10 items). Five of the 10 items are reverse scored, as they express a preference for well-known places and discomfort with unfamiliar ones (e.g., “When I see a new road, I avoid taking it because I don’t know where it goes”). Each item is rated on a 6-point Likert scale (1 = not at all to 6 = very much), and the sum is calculated after the five negative items have been reversed (score 10–60; Cronbach's *α* = 0.84, current sample).

*Sense of Direction and Spatial Representation scale* (SDSR; De Beni et al., 2014; Pazzaglia & Meneghetti, 2017). The scale measures an individual’s self-reported sense of direction (e.g., “I think to have good sense of direction” [item 1]; 1–5; these items resemble Hegarty et al., 2002 scale; e.g., “my sense of direction is very good” [item 4], 1–7); further, it assesses the usage of cardinal points, and preferences for survey or route/landmark-based modes to orient in the environment (13 items). Each item is rated on a 5-point Likert scale (1 = not at all to 5 = very much), and the sum is calculated (score 13–65; Cronbach’s *α* = 0.84 in the current sample). This scale was used because validated in the Italian context showing good psychometrical properties (original sample Cronbach’s α = 0.84, test retest *r* = 0.78, correlation with SBSOD by Hegarty et al., 2002; *r* = 0.57, for details see Pazzaglia & Meneghetti, 2017).

#### Session 2: learning from navigation and recall

#### Path learning phase

**Fig. 1:**
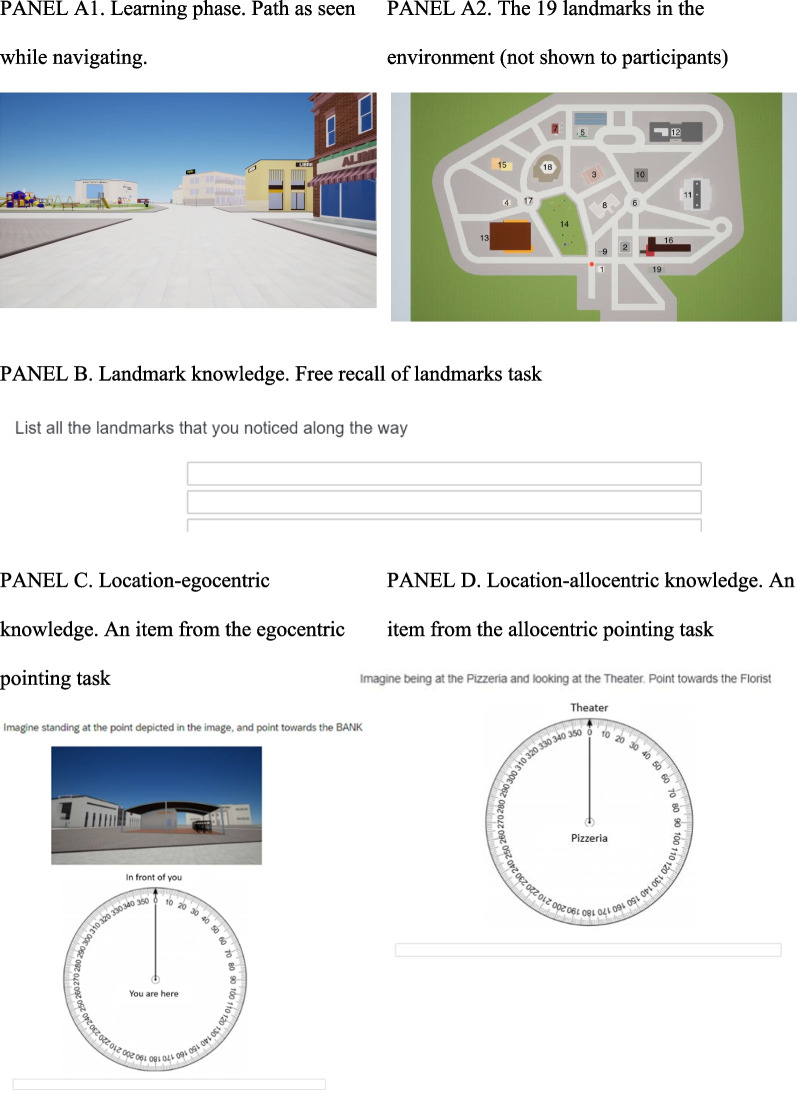

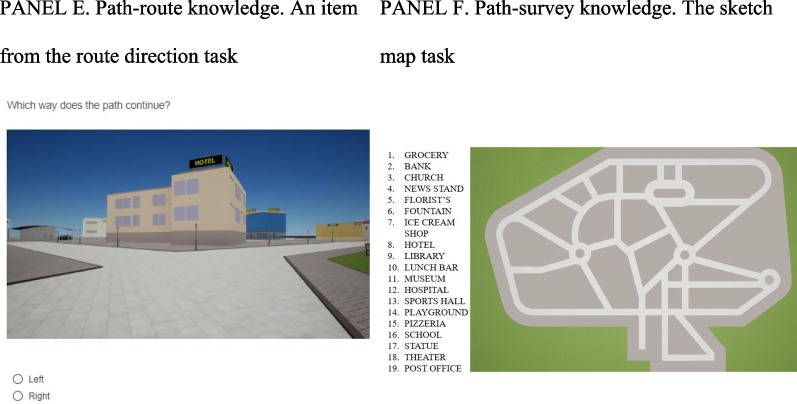
Virtual city with the path to learn and examples of each recall task used to test the different types of environmental knowledge. *Note.* These stimuli were adapted from the original Italian version. List of landmarks as encountered in the path: 1. grocery; 2. bank; 3. church; 4. newsstand; 5. florist; 6. fountain; 7. ice-cream shop; 8. hotel; 9. library; 10. lunch bar; 11. museum; 12. hospital; 13. sports hall; 14. play park; 15. pizzeria; 16. school; 17. statue; 18. theatre; 19. post office

A video of a path about 1 km long in a virtual city (modeled with Rhino, Unreal Engine Version 4.21), as seen from a first-person perspective (eye height of 160 cm, camera set with a horizontal field of view of 90°) was used in the learning phase. Participants watched the video twice on a laptop, with each presentation lasting about 4 min (4 m/s walking speed). During the video presentation, 19 landmarks depicting common city buildings were presented (see Fig. 1, panel A1; for a map of the environment with the path and landmarks, see Fig. 1, panel A2; the map was not shown to participants).

#### Recall phase

*Free recall of landmarks task – Landmark knowledge.* This involved participants writing as many landmarks as they could recall, in any order (see Fig. 1, panel B). One point was awarded for each landmark correctly recalled and the sum was calculated (score 0–19).

*Egocentric pointing task – Location-egocentric knowledge.* In this task, participants were asked to imagine standing in front of a landmark shown by a screenshot and then point in the direction of another landmark (example: screenshot of the lunch bar—“Imagine standing here, and point to the bank”). For each item, the question was written at the top of a page showing a screenshot of a landmark, with a graduated circle underneath. The answer was given by writing the degrees of the angle corresponding to the direction (see Fig. 1, panel C). There were 6 items in all, plus one for familiarization. The mean of the minimum angles between each participant’s response and the correct direction was considered (0–180°).

*Allocentric pointing task – Location-allocentric knowledge.* In this case participants were asked to imagine standing at a given landmark, facing another, and pointing to a third (example: “Imagine standing at the pizzeria, and looking at the theater, the point to the florist’s”; see Fig. 1, panel D). For each item, the question was written at the top of a page and the answer given by writing the degrees of the angle corresponding to the direction. There were 6 items in all, plus one for familiarization. The mean of the minimum angles between each participant’s response and the correct direction was considered (0–180°).

*Route direction task – Path-route knowledge.* Participants were shown a screenshot representing a crossroads along the previously-learned path, and asked to choose which way to go to repeat the route they had taken, choosing between two options (left or right; right or straight on; left or straight on). There were 7 items, plus one for familiarization. One point was awarded for each correctly identified direction to take along the path (score range 0–7).

*Sketch map task – Path-survey knowledge.* This consisted in placing the 19 landmarks as seen along the path. The landmarks are shown on a list in alphabetical order and participants have to write their corresponding number on a sketch map of the environment showing all the roads (see Fig. 1, panel F). For scoring purposes, participants were awarded 0 points when they did not position the landmark correctly (within a gray area formed by the intersection of roads), half a point when they located the landmark within the right gray area, but not precisely in its correct position, or 1 point when the landmark was placed in exactly the right position (for details, see ). The final score was obtained from the sum of the landmarks more or less correctly positioned (range 0–19); as a control, the number of landmarks placed on the map (irrespective of their location; range 0–19) was also computed. The experimenter scored all sketch maps, and a second judge scored a random sample of 60 sketch maps. The two independent scores correlated closely (*r* = 0.96), so the analyses were run on the scores returned by the first judge (the experimenter).

### Procedure

Participants signed an informed consent form and individually attended two remote sessions lasting 45 min each. In both sessions, the experimenter met participants on the Zoom platform, provided a Qualtrics link, and remained connected to them (creating a lab-like condition). Both the participants and the experimenter had their cameras and microphones on throughout the sessions. The participants completed the experiment by sharing their screen with the experimenter. In the first session, participants completed the visuospatial tasks (Jigsaw Puzzle Test, short Mental Rotation Test, and short Object Perspective Taking test) and the questionnaires (spatial self-efficacy, Attitude toward Orientation tasks, Sense of Direction and Spatial Representation scale) in random order. During a second session, participants first learned the path by watching the video twice, then they completed the free recall of landmarks, egocentric pointing (with randomly presented items), allocentric pointing (with randomly presented items), route direction (with randomly presented items), and sketch map tasks, all tasks in random order. When the sketch map task appeared in Qualtrics, participants followed a link that redirected them to a Google Jamboard file in which to perform the task.

### Data analysis

**Fig. 2:**
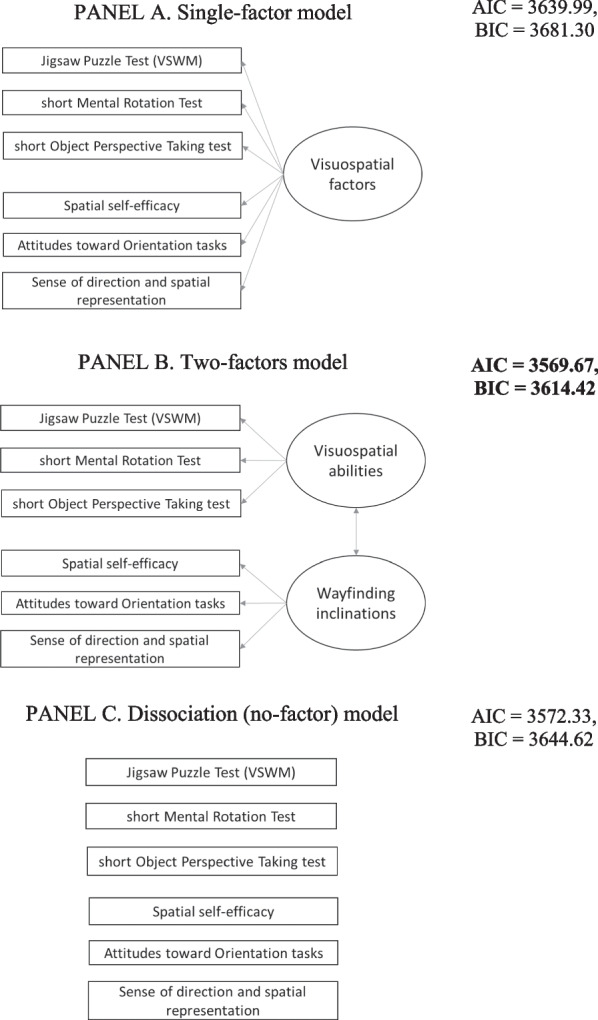
All tested factorial structures of visuospatial factors, and AIC and BIC for model selection. *Note.* Covariance not shown in the graphs but considered in the models. Two-factors model (Panel **b**) was chosen based on AIC and BIC criteria (in bold type). **a** Single-factor model. **b** Two-factors model. **c** Dissociation (no-factor) model

Data were analyzed using R (R Core Team, 2020). For descriptive purposes means and standard deviations and correlations were computed first. Then the factor composition of visuospatial abilities and wayfinding inclinations with environmental knowledge was assessed using confirmatory factors analyses. Concerning visuospatial factors (see Fig. 2), we examined a two-factor model (Fig. 2, panel b) with one factor for visuospatial abilities (Jigsaw Puzzle Test—VSWM, the short Mental Rotations Test and the short Object Perspective-taking Test) and the other for wayfinding inclinations (Sense of Direction and Spatial Representation scale, Attitudes toward Orientation Tasks scale, and Spatial Self-Efficacy; as in Meneghetti et al., 2021). We also examined a single-factor model, considering all visuospatial factors together as one factor (Fig. 2, panel a), and a dissociation (no-factor) model in which each visuospatial task and questionnaire were considered separately (also considering covariances between measures; Fig. 2, panel c).

Concerning environmental knowledge (see Fig. 4), we examined: a single-factor model (Hegarty et al., 2006; Fig. 4, panel a); a model considering landmark, egocentric/route and allocentric/survey knowledge (Wiener et al., 2009; Fig. 4, panel b); a model considering landmark, location, and path knowledge (Claessen & van der Ham, 2017; Fig. 4, panel c); a model considering the dissociation between all different types of knowledge, i.e. each recall task being considered separately, albeit with the covariance between them (van der Ham et al., 2020; Fig. 4, panel c).

Then, a structural equation model was used to investigate the relationship between participants’ visuospatial factors and environmental knowledge (considering the compositions of the factors emerged in the previous step). We hypothesized a model in which the visuospatial abilities and inclinations factor(s) predicted the environmental knowledge factor(s). Given the well-known debate on gender-related differences in environmental knowledge (e.g., Nazareth et al., 2019), we also examined whether the structural equation model differed between men and women using a multiple-group approach aimed at detecting any difference in men and women in the relationship between variables hypothesized. Note the intent was not to investigate the effect of gender on environment knowledge through the mediation of visuospatial abilities and inclinations factor(s) (as in Miola et al., 2021a, 2021b; Pazzaglia et al., 2018); however, this mediation is replicable with our data (see Additional file 1).

Concerning the statistical indexes used for models, the Akaike Information Criterion (AIC; Wagenmakers & Farrell, 2004; smaller is good) and the Bayesian Information Criterion (BIC; Schwarz, 1978; smaller is good) were used to compare the models (Burnham & Anderson, 2004) to define the better factor composition of visuospatial abilities and inclinations. The following were used as fit indices of the selected confirmatory factor models and of the structural equation model: the root-mean-square error of approximation (RMSEA; ≥ 0, small is good), the standardized root mean square residual (SRMR; ≥ 0, small is good), the comparative fit index (CFI; [0,1], large is good), and the nonnormed fit index (NNFI; which can fall outside [0,1], large is good). Maximum likelihood was used to estimate the parameters of the models. The confirmatory factor analyses and the structural equation modeling procedure were run using the “lavaan” package (Rossel, 2012).

## Results

Table 1 shows the means and standard deviations of participants’ visuospatial factors, with the correlations between all the variables (only a *p* < 0.001 was considered significant, given the multiple comparisons).

**Table 1:** Means, standard deviations and correlations between all variables

	*1*	*2*	*3*	*4*	*5*	*6*	*7*	*8*	*9*	*10*	*11*	*12*
1. Age												
2. Visuospatial Working Memory (Jigsaw Puzzle Test)	− .13^*^											
3. short Mental Rotations Task	− .07	.43^***^										
4. short Object Perspective-taking Test	.10	− .34^***^	− .38^***^									
5. Spatial Self-efficacy	− .02	.21^**^	.19^**^	− .01								
6. Attitude toward Orientation Task	.09	.19^**^	.29^***^	− .17^*^	.58^***^							
7. Sense of Direction and Spatial representation	− .01	.23^***^	.29^***^	− .17^*^	.67^***^	.70^***^						
8. Free recall of landmarks (landmark knowledge)	.03	.17^*^	.12	− .19^**^	.16^*^	.12	.11					
9. Egocentric pointing (location-egocentric knowledge)	.01	− .29^***^	− .23^***^	.30^***^	− .21^**^	− .19^**^	− .24^***^	− .16^*^				
10. Allocentric pointing (location-allocentric knowledge)	.07	− .19^**^	− .19^**^	.18^**^	− .25^***^	− .26^***^	− .22^***^	− .30^***^	.20^**^			
11. Route direction (path-route knowledge)	− .08	.15^*^	.14^*^	-.09	.06	.21^**^	.15^*^	.14^*^	− .16^*^	− .27^***^		
12. Sketch map (path-survey knowledge)	− .03	.31^***^	.27^***^	− .22^***^	.41^***^	.38^***^	.43^***^	.36^***^	− .38^***^	− .45^***^	.32^***^	
*M*	25.48	12.33	5.13	40.69	31.27	39.63	36.84	11.14	61.51	74.85	5.74	5.55
*SD*	6.06	2.63	2.64	36.68	5.41	8.08	7.55	2.98	22.98	30.21	1.16	4.08

*N* = 270; ^*^p < 0.05; ^**^0.16, *p* < 0.01, ^***^0.19, *p* < 0.001 (the latter in bold type, the ones considered significant given multiple comparisons)

Egocentric and allocentric pointing, and sketch map drawing correlated with all visuospatial abilities and wayfinding inclinations considered. Free recall of landmarks correlated with perspective-taking abilities. Route direction correlated with the attitude towards orientation task. The sketch map drawing and allocentric pointing tasks correlated with each other, and with all the other recall tasks used, while the other tasks did not correlate with one another. The random presentation of the tasks mainly did not present an effect on task accuracy 1.

### Factor composition for visuospatial factors

**Fig. 3:**
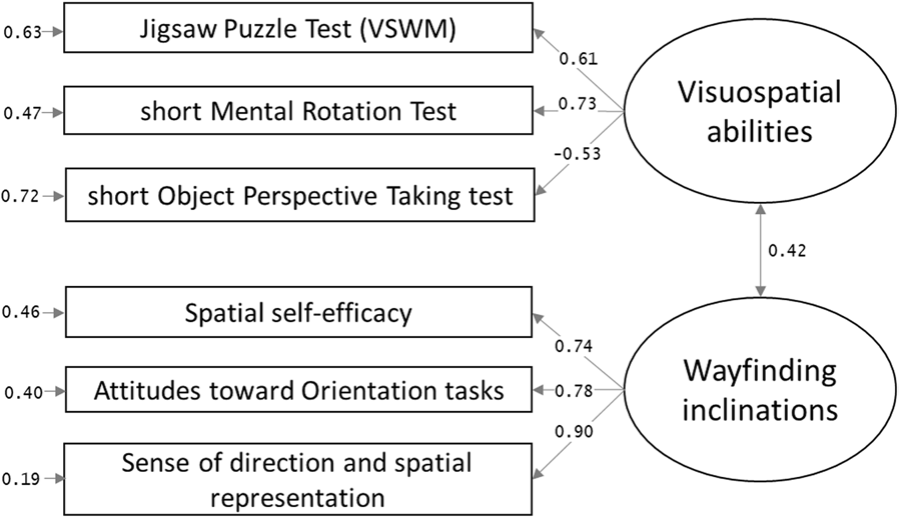
Confirmatory factor analysis with standardized factor loadings for visuospatial factors

The confirmatory factor analyses showed that the AIC and BIC of the two-factor model (Fig. 2, panel b), considering visuospatial abilities (factor 1) and inclinations (factor 2), were lower (AIC = 3569.67, BIC = 3614.42) than that of the single-factor model (Fig. 2, panel a; AIC = 3639.99, BIC = 3681.30) or the dissociation (no-factor) model (Fig. 2, panel c) considering all visuospatial measures singularly (AIC = 3572.33, BIC = 3644.62). The two-factor model was the best model. It was adequate in terms of the factor composition, *χ*2 (8) = 13.34, *p* = 0.101, RMSEA = 0.05, SRMR = 0.04, CFI = 0.99, NNFI = 0.98. All loadings were > 0.52 (in absolute values). See Fig. 3.

### Factor composition for environmental knowledge

**Fig. 4:**
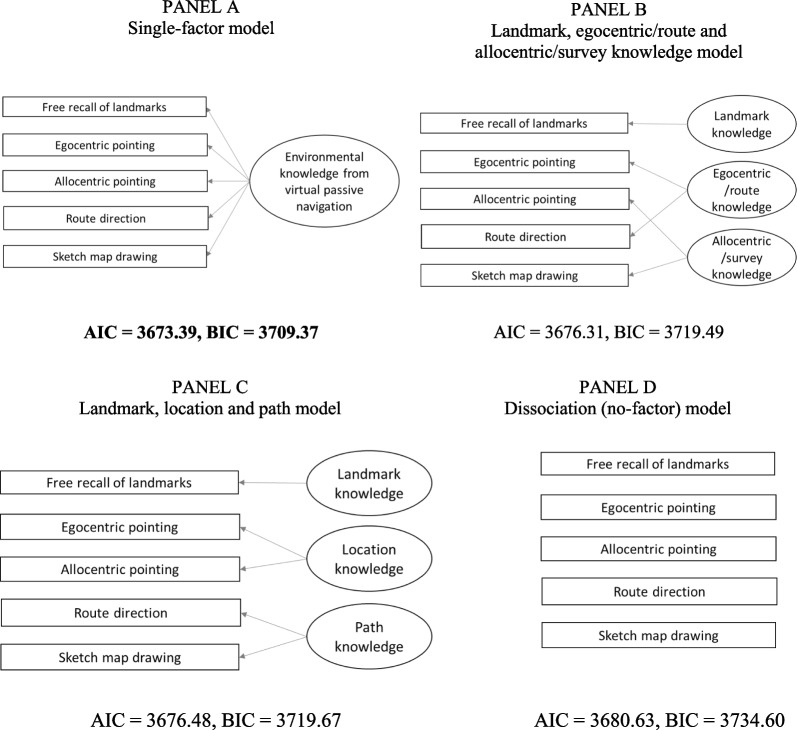
All tested factorial structures of environmental knowledge, and AIC and BIC for model selection. *Note.* Covariance not shown in the graphs but considered in the models. Single-factor model (Panel **A**) was chosen based on AIC and BIC criteria (in bold type). **a** Single-factor model. **b** Landmark, egocentric/route and allocentric/survey knowledge model. **c** Landmark, location and path model. **d** Dissociation (no-factor) model

**Fig. 5:**
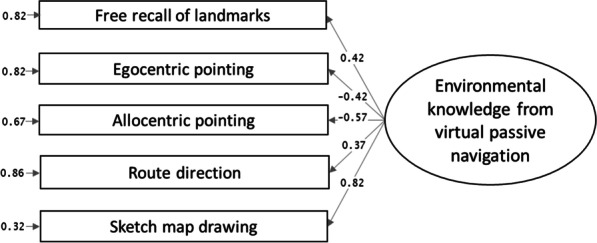
Confirmatory factor analysis with standardized factor loadings for environmental knowledge gained from navigating a VE online

Concerning the comparison between the hypothesized models from literature, the confirmatory factor analyses showed that the AIC of the single-factor model (Fig. 4, panel a; AIC = 3673.39, BIC = 3709.37) was lower than that of the model that considered all the recall tasks separately (dissociation model, Fig. 4, panel d; AIC = 3680.63, BIC = 3734.60) and the models that considered three factors (Fig. 4, panel b, landmark, egocentric/route, allocentric/survey knowledge; AIC = 3676.31, BIC = 3719.49; Fig. 4, panel c, landmark, location, and path knowledge, AIC = 3676.48, BIC = 3719.67). Therefore, the single-factor model was the best model. The single factor model was adequate regarding the factor composition, χ2(5) = 2.76, *p* = 0.736, RMSEA = 0.001, SRMR = 0.02, CFI = 1.00, NNFI = 1.03. All loadings were ≥ 0.37 (in absolute values). To be specific, the sketch map drawing task had the highest load (0.82) on the environmental knowledge factor, followed by the allocentric pointing task (− 0.57), and then the route direction task with the lowest load (0.37). See Fig. 5.

### Structural equation modelling

**Fig. 6:**
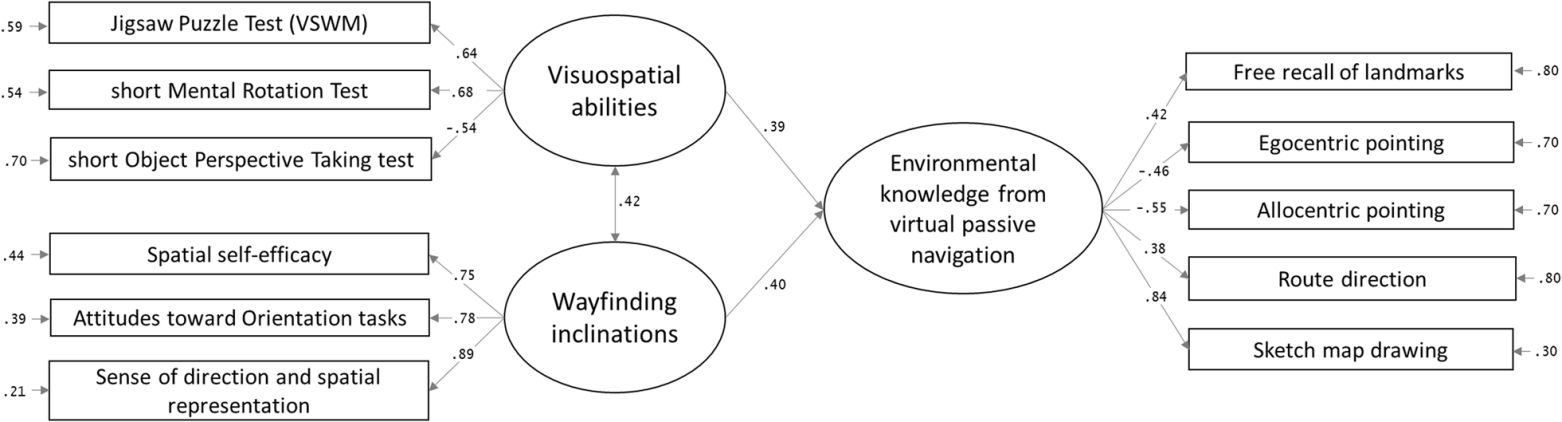
Structural model with standardized coefficients. *Note for *Figs. 5 and 6. In the egocentric and allocentric pointing tasks, the scores coincide with the degrees of error

The fit of the model considering participants’ visuospatial abilities and inclinations (two-factor model) predicting environmental knowledge (one-factor model) was adequate, *χ*2(41) = 55.96, *p* = 0.060, RMSEA = 0.04, SRMR = 0.05, CFI = 0.98, NNFI = 0.97. The total variance accounting for the environmental knowledge factor was 44%. Specifically, the variance accounting for free recall of landmarks was 17%, for egocentric pointing it was 21%, for allocentric pointing 30%, for route direction 15%, and for sketch map drawing 70%. The structural model and factor composition are shown in Fig. 6, and the coefficients with *p* values and confidence intervals in Table 2. Both the visuospatial abilities factor and the wayfinding inclinations factor were significantly related (with similar coefficients) to the environmental knowledge factor for knowledge gained from a VE.

**Table 2:** Coefficient of the structural model

	Estimates	Standardized estimates	*p*	95% CI
Visuospatial abilities → Environmental knowledge	.25	.39	.001	[.10, .40]
Wayfinding inclinations → Environmental knowledge	.22	.40	< .001	[.11, .34]
Visuospatial abilities ↔ Wayfinding inclinations	.20	.42	< .001	[.10, .29]

Concerning gender using a multiple-group approach, results showed that the model in which the parameters were constrained to be equal between the genders did not differ (*χ*2(10) = 15.33, *p* = 0.121) from the model in which they were not. This would suggest that the model and its relationships did not differ significantly between men and women.

## Discussion and conclusion

A relevant issue to investigate in spatial navigation concerns the spatial knowledge gained after navigating a VE (Hegarty et al., 2006; Meneghetti et al., 2021). To gain a comprehensive picture of an individual’s spatial knowledge it is important to assess several domains, such as landmark, egocentric and allocentric location, route and survey path knowledge (van der Ham et al., 2020). Spatial knowledge gained from navigation has mostly been measured by testing only some of these various domains of knowledge, however, and more information is needed about the relationship between them (van der Ham et al., 2020; Muffato et al., 2022). Various human factors, such as visuospatial abilities and wayfinding inclinations, have been found to support environmental knowledge (Ishikawa, 2022; Meneghetti et al., 2021), but how they relate to the different types of environmental knowledge gained after passively learning a VE has yet to be investigated. Learning from passive navigation may result in reduced environmental knowledge (Chrastil & Warren, 2015). However, it may be sustained from visuospatial factor(s), and therefore, this issue deserved investigation.

A sample of individuals was assessed via an online link on VSWM, mental rotation and perspective-taking (visuospatial abilities), and on sense of direction, attitudes toward orientation tasks, and spatial self-efficacy (wayfinding inclinations). Then they passively navigated a path in a VE and were tested with a free recall landmark task (assessing landmark knowledge), egocentric and allocentric pointing tasks (assessing location-egocentric and -allocentric knowledge), and a route direction and a sketch map drawing task (assessing path-route and survey knowledge, respectively).

First, we examined the factorial structure of the visuospatial measures and environmental knowledge, running confirmatory factor analyses. The distinction between visuospatial abilities (grouping the tasks that assess these abilities from basic processing to higher cognitive levels abilities; Hegarty et al., 2006; Pazzaglia et al., 2018) and wayfinding inclinations (grouping questionnaires that assess spatial orientation, self-efficacy, and attitude toward orientation in the environment) was confirmed, corroborating previous findings (e.g., Meneghetti et al., 2021). This is further confirmation of the validity to distinguish human factors related to environmental knowledge mainly in two factors, that is, abilities and inclinations, that can be considered in further research.

Regarding the new investigations in the present study, we examined all hypotheses for the factorial structure of the environmental knowledge gained after navigating passively a VE: a dissociation between the domains (van der Ham et al., 2020); a distinction between landmark, location, and path knowledge (Claessen & van der Ham, 2017); a distinction between three types of knowledge (landmark, egocentric/route, and allocentric/survey knowledge; Wiener et al., 2009); and a single-factor composition (Hegarty et al., 2006).

We found that environmental knowledge, as tested with our tasks after passive navigation learning, relies on a single factor; in other words, all the domains of spatial knowledge (landmark, egocentric/allocentric location, paths route/survey form part of a single latent factor. It is important to note, however, that each task loaded the environmental knowledge factor differently: the route direction task (testing path-route knowledge) had the lowest load, and the sketch map drawing task (testing survey-path knowledge) the highest, with the allocentric pointing task (testing allocentric location knowledge) in between. The descriptive statistics (Table 1) show that, on average, participants’ answers were most accurate in the route direction task, and less so in the allocentric pointing and sketch map drawing tasks. Performance in the latter two tasks correlated with all the other environmental recall tasks. Taken together, these results suggest that the allocentric pointing and sketch map drawing tasks are useful for assessing navigation ability. The characteristics of the two tasks may explain these results, as they both involve managing information in the mental representation from a different view from the one adopted in the learning phase (Muffato et al., 2020). Our study was novel in that it focused on different types of navigation knowledge acquired by desktop VE navigation, considered passive navigation learning. Although most previous studies have focused on active navigation, they have not necessarily considered all types of environmental knowledge. Some studies have focused on a single type of knowledge to answer a specific research question, such as assessing survey knowledge after navigation learning (Miola et al., 2021a, 2021b). Further studies should compare passive and active navigation learning (e.g., Chrastil & Warren, 2015) and examine the resulting structure of environmental knowledge to provide a more complete picture.

After establishing the factor composition of visuospatial factors (best represented by the two-factor model: visuospatial abilities, wayfinding inclinations) and environmental knowledge (best represented by the single-factor model including performance in all recall tasks), the main aim of the study was to investigate whether the former related to the latter, even after learning from passive navigation. The results of our SEM model showed that visuospatial abilities and wayfinding inclinations both predicted the environmental knowledge gained from navigating a VE. Looking at the beta values of our SEM model, each factor seems to contribute to environmental knowledge to a similar extent. This result differs from previous lab findings on learning from navigation in a desktop VE, when visuospatial abilities had a greater role than wayfinding inclinations (Hegarty et al., 2006; Meneghetti et al., 2021; Miola et al., 2021a, 2021b; Pazzaglia et al., 2018). In the present study, the VE was navigated passively from an online video, and it showed a similar involvement of visuospatial abilities and wayfinding inclinations. In contrast, previous findings used active virtual navigation (for instance, with a joystick, Meneghetti et al., 2021; Pazzaglia et al., 2018; with a keyboard, Hegarty et al., 2006). However, note that Hegarty et al. (2006) also included a passive videotape condition, but it loaded onto the same factor as the virtual navigation did. This paves the way for future studies considering learning modalities (active vs. passive), environmental knowledge, and relationships with human factors. Then, note that the finding of a similar contribution of visuospatial abilities and inclinations in passive VE navigation suggests that people with more positive wayfinding inclinations may have more confidence in approaching spatial navigation tasks in general, even when passively learning online. Although visuospatial inclinations reflect people’s self-reported ability to navigate in real-life situations using body-based cues, which are absent in online VE, people who provide higher ratings may generally be more engaged in spatial tasks and may learn more effectively even through passive navigation. The relationship between wayfinding inclinations (based on real-life situations) and navigation learning (simulated through desktop projection without directly involving physical movement) supports the future use of VEs on desktops as useful for examining the relationship with human factors. Alternatively, visuospatial abilities may be less strongly related to environment learning performance in our experimental conditions because they are higher-level abilities usually tested with paper and pencil tasks (Hegarty et al., 2006), and the online test may not fully reflect individual performance. The present study offers new insight on passive VE navigation, suggesting that human factors can affect environmental knowledge even when it is learned and tested online. Visuospatial abilities and wayfinding inclinations both seem important in supporting our performance when we face the challenge of learning spatial information from an online tool showing a path. This novel aspect deserves further investigation, comparing online active and passive VE navigation with VE navigation in a laboratory setting, or with real-life navigation. Not having drawn such comparisons here limits any generalization of our results to navigation in a general sense.

Some considerations can also be made on each type of spatial knowledge task in relation to visuospatial factors, based on the correlations and the variance explained in the SEM models. It seems that, after learning from navigation (based on a route view), a task assessing recall from a survey view (the sketch map task), and tasks that involve the active use of mental representations to judge directions (egocentric and allocentric pointing tasks) prompted the greatest involvement of visuospatial abilities and wayfinding inclinations, and the task assessing recall from a route view (the route direction task) triggered only a marginal involvement of visuospatial factors (as suggested by Meneghetti et al., 2021). It is also worth noting that the free landmark recall task was found to correlate with perspective-taking abilities, and with the allocentric location pointing and sketch map drawing tasks. It may be that, when recalling landmarks, people also retrieve location and path knowledge because they associate landmarks with their positions. This is an aspect that deserves further investigation.

Gender is also worth considering. We found that the relationship between visuospatial abilities and inclinations and environmental knowledge was similar for both men and women. This means that women with higher abilities and inclinations can benefit from them similarly to men. While women with lower visuospatial abilities and inclinations may have lower environmental knowledge than men (in line with models inserting gender as an initial predictor, Miola et al., 2021a, 2021b; Pazzaglia et al., 2018; see Additional file 1), the finding that gender does not affect the entity of the relationship between visuospatial factors and environmental knowledge highlights the importance of promoting and enhancing these human factors in environment knowledge acquisition, regardless of gender.

Overall, these results contribute to enlarging the theoretical frame regarding spatial knowledge gained from passively navigating a VE and the different types of knowledge acquired in relation to human factors (in terms of the several and simultaneous effects of visuospatial factors). The present study offers fresh insight on the similar involvement of visuospatial abilities and wayfinding inclinations in the spatial knowledge acquired and tested using an online modality. This corroborates the idea that it is important to take visuospatial abilities and wayfinding inclinations into account (Hegarty et al., 2006; Meneghetti et al., 2021), even in passive navigation.

Although it offers some important insight, this study has some other limitations (as well as the lack of a comparison between learning from navigation online as opposed to desktop VE or real-life settings, and the lack of comparison of active/passive navigation). They mainly concern the tasks used to measure each type of environmental knowledge. Although they had already been used in other studies (Muffato et al., 2022; van der Ham et al., 2020), they might not fully represent the type of knowledge considered. Our results may therefore depend on the characteristics of the tasks rather than on the types of knowledge. This issue can be clarified by administering more than one task for each type of knowledge. Future studies could include more tasks assessing the same type of knowledge in different ways (using landmark recognition as well as free recall of landmarks to test landmark knowledge, for instance, or both a route direction task and a route repetition task to test path-route knowledge). Such future studies could then examine whether different levels of recall accuracy and degrees of involvement of visuospatial factors actually reflect differences in the difficulty of acquiring certain types of knowledge, or are attributable to the types of task involved. In fact, there is evidence to suggest that people’s navigation performance is influenced not only by the quality of their mental representations of an environment, but also by the instructions and characteristics of the task used to test it (Boone et al., 2019). Another limitation to consider is the type of VE we used. The environment was a city with few buildings that served as landmarks, which somewhat resembles a vista space. In vista spaces, spatial information is visible from a single point of view (Montello, 1993). This differs from the actual navigation in environmental spaces in which space is larger than the learner’s body, demanding locomotion to gain a full experience of them. As such, landmarks’ visibility can be a crucial factor in navigation learning. Future studies should aim to replicate these findings using more realistic city contexts and to consider landmark visibility manipulations, such as adding boundaries to restrict participants to viewing a single landmark from any given point on the path (Meilinger et al., 2016) for a clearer understanding of navigation learning.

To conclude, this study has shown that different types of spatial knowledge can be formed after learning from passive online VE navigation. Gaining such knowledge requires a degree of involvement of human factors, such as visuospatial abilities and positive wayfinding inclinations.

## Supplementary Information

**Additional file 1**. Supplementary material.

## Data Availability

The data that support the findings of this study are openly available in figshare at https://doi.org/10.6084/m9.figshare.20392470.v1

## Footnotes

1 Due to the random order in which the tasks were completed, we checked whether there was any difference in the free recall of landmarks performance based on the order of presentation, given that in the other tasks, landmarks are presented to participants. We found no difference in performance among 106 different presentation orders, F(105,164) = 0.86, *p* = .797. However, when checking the difference between the performance of the free recall task after having completed the sketch map task, the task that provides explicitly the list of landmarks, we observed a significant performance difference, t(267.93) = 4.71, *p* < 0.001, Cohen’s *d* = .57 (completing free recall before the map task, *M* = 10.37, *SD* = 3.00, after *M* = 12.00, *SD* = 2.71).
