# Supplementary material for: Investigating the different domains of environmental knowledge acquired from virtual navigation and their relationship to cognitive factors and wayfinding inclinations
Source: Cogn Res Princ Implic. 2023 Aug 2;8:50. doi: 10.1186/s41235-023-00506-w (PMC10397164; doi:10.1186/s41235-023-00506-w)

Supplementary materials

Investigating the different domains of environmental knowledge acquired from virtual navigation and their relationship to cognitive factors and wayfinding inclinations

As gender is known to play a role in spatial cognition (Nazareth et al., 2019), we investigated whether it has a role in affecting environmental knowledge through the mediation of visuospatial factors. To do this, we ran a mediation model similar to those used in previously studies (Miola et al., 2021; Pazzaglia et al., 2018) and replicated the results using our sample. We found that visuospatial abilities (std β= .15, p = .002) and wayfinding inclinations (std β= .12, p = .002) mediate the relationship between gender and environmental knowledge, as shown in Figure S1. The model had acceptable fit indexes: $\chi 2(50) = 83.26$, p = .002, RMSEA = 0.05, SRMR = 0.07, CFI = 0.95, NNFI=0.94.

It should be noted that we replicated the findings newly considering various types of tasks as a single factor and considering learning the environment from passive online VE (both Miola et al., 2021 and Pazzaglia et al., 2018 were active VE navigation, which involved navigating with a joystick).

Figure S1. Mediation model of visuospatial factors between gender and environmental knowledge

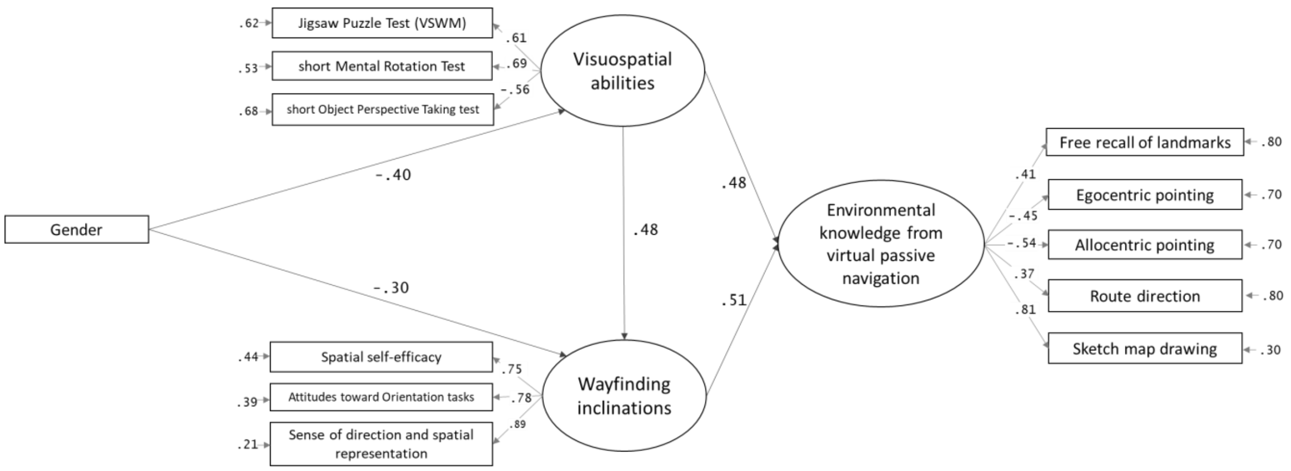

Supplement: Supplementary file 1 — Additional file 1. Supplementary material. [file 41235_2023_506_MOESM1_ESM.pdf]
